# Assessing Medical Students’ Perceptions of AI-Integrated Telemedicine: A Cross-Sectional Study in Romania

**DOI:** 10.3390/healthcare13090990

**Published:** 2025-04-24

**Authors:** Florina Onetiu, Melania Lavinia Bratu, Roxana Folescu, Felix Bratosin, Tiberiu Bratu

**Affiliations:** 1 Doctoral School, “Victor Babes” University of Medicine and Pharmacy, Eftimie Murgu Square 2, 300041 Timisoara, Romania; florina.onetiu@umft.ro; 2 Department of Neurosciences, “Victor Babes” University of Medicine and Pharmacy, Eftimie Murgu Square 2, 300041 Timisoara, Romania; 3 Discipline of Family Medicine, “Victor Babes” University of Medicine and Pharmacy, Eftimie Murgu Square 2, 300041 Timisoara, Romania; 4 Multidisciplinary Research Center for Infectious Diseases, “Victor Babes” University of Medicine and Pharmacy, Eftimie Murgu Square 2, 300041 Timisoara, Romania; felix.bratosin@umft.ro; 5 Discipline of Plastic Surgery, “Victor Babes” University of Medicine and Pharmacy, Eftimie Murgu Square 2, 300041 Timisoara, Romania; tiberiu.bratu@umft.ro

**Keywords:** telemedicine, Artificial Intelligence, medical students, healthcare technology, perception, Romania, cross-sectional study, education

## Abstract

Background and Objectives: The rapid advancement of Artificial Intelligence (AI) has driven the expansion of telemedicine solutions worldwide, enabling remote diagnosis, patient monitoring, and treatment support. This study aimed to explore medical students’ perceptions of AI in telemedicine, focusing on how these future physicians view AI’s potential, benefits, and challenges. Methods: A cross-sectional survey was conducted among 161 Romanian medical students spanning Years 1 through 6. Participants completed a 15-item questionnaire covering demographic factors, prior exposure to AI, attitudes toward telemedicine, perceived benefits, and concerns related to ethical and data privacy issues. A questionnaire on digital health acceptance was conceived and integrated into the survey instrument. Results: Out of 161 respondents, 70 (43.5%) reported prior telemedicine use, and 66 (41.0%) indicated high familiarity (Likert scores ≥ 4) with AI-based tools. Fifth- and sixth-year students showed significantly greater acceptance of AI-driven telemedicine compared to first- and second-year students (*p* = 0.014). A moderate positive correlation (r = 0.44, *p* < 0.001) emerged between AI familiarity and telemedicine confidence, while higher data privacy concerns negatively affected acceptance (*β* = −0.20, *p* = 0.038). Gender differences were noted but did not reach consistent statistical significance in multivariate models. Conclusions: Overall, Romanian medical students view AI-enhanced telemedicine favorably, particularly those in advanced academic years. Familiarity with AI technologies is a key driver of acceptance, though privacy and ethical considerations remain barriers. These findings underline the need for targeted curricular interventions to bolster AI literacy and address concerns regarding data security and clinical responsibility. By proactively integrating AI-related competencies, medical faculties can better prepare students for a healthcare landscape increasingly shaped by telemedicine.

## 1. Introduction

Telemedicine, broadly defined as the remote delivery of healthcare services using digital communication, has emerged as a vital component of contemporary medical practice [1,2,3]. Advances in internet connectivity, mobile technologies, and health informatics have collectively enabled healthcare professionals to extend their reach beyond traditional clinical settings [4]. In tandem with this growth, Artificial Intelligence (AI) systems have become increasingly prominent, offering capabilities such as automated diagnostic support, patient triage, and personalized treatment planning [5,6,7].

Integrating AI into telemedicine holds significant promise. From reducing diagnostic errors to improving patient monitoring, AI-driven teleconsultations can streamline patient–provider interactions and potentially mitigate resource constraints [8,9]. However, the acceptance of these technologies is not guaranteed, particularly if end-users harbor reservations about data confidentiality, algorithmic transparency, or the perceived reliability of automated diagnostic tools [10,11].

Medical students, as future practitioners, represent a critical demographic in understanding the trajectory of AI-supported telemedicine [12,13]. Their attitudes can shape the adoption and responsible use of these technologies. While clinical rotations and hands-on experiences traditionally define medical education, the increasing emphasis on virtual platforms necessitates a parallel focus on telemedicine competencies [14].

Recent global shifts, including pandemic-driven disruptions, have accelerated the adoption of remote healthcare services [15,16,17]. Yet the depth of medical students’ exposure to AI-based telemedicine varies considerably [18]. This discrepancy underscores the need for empirical data on how these emerging physicians perceive the efficacy, safety, and ethical soundness of AI-enhanced remote care [19].

This cross-sectional study aims to (1) gauge medical students’ perceptions of AI-powered telemedicine, (2) compare acceptance levels across different academic years, and (3) identify key facilitators and barriers influencing attitudes. We hypothesize that students with higher academic seniority and greater exposure to AI applications will exhibit a more positive attitude toward AI-integrated telemedicine. Furthermore, we propose that concerns about patient confidentiality, malpractice liability, and technological reliability may moderate overall acceptance [20]. The findings can inform medical education strategies by highlighting specific content areas—such as data security, ethical considerations, and hands-on training with AI tools—that may enhance preparedness for a rapidly evolving healthcare ecosystem.

## 2. Materials and Methods

### 2.1. Study Design and Setting

This cross-sectional study was conducted at Victor Babes University of Medicine and Pharmacy Timisoara, Romania, over a twelve-month period. Ethical approval was granted by the institutional review board of Victor Babes University of Medicine and Pharmacy Timisoara. Participation was voluntary, and the target population included all medical students enrolled in Years 1 to 6. To be eligible, participants had to be at least 18 years old and have no prior formal clinical practice experience (beyond standard academic requirements).

An a priori power analysis (G*Power 3.1) for detecting a medium correlation (ρ = 0.30) between AI familiarity and acceptance at α = 0.05 and 80% power indicated a minimum of 138 respondents; recruiting 161 students therefore exceeded the target by 17%, ensuring adequate power for both correlation and regression analyses. The final sample included 161 students (57 in Years 1–2, 49 in Years 3–4, and 55 in Years 5–6), who provided consent to participate.

Recruitment was conducted in person after courses. Students were notified of this study’s purpose—to understand perceptions of AI-driven telemedicine—and were informed about data confidentiality. Although telemedicine is not routinely practiced within the university setting, students may have encountered it elsewhere through internships or personal healthcare experiences. At the time of the survey, Victor Babeș University did not offer any stand-alone telemedicine or AI-focused modules—neither as core requirements nor as electives—so students’ exposure to these topics derived solely from informal sources such as clinical placements, extracurricular workshops, or personal use of digital health apps.

Data collection involved a paper-based format. Paper-based versions were administered following lecture sessions. Students were informed that participation was voluntary, and consent forms were obtained prior to survey completion. All questionnaires were anonymized using numeric codes, and no personal identifiers were collected in the final dataset. Participants were free to withdraw at any point without academic repercussions.

### 2.2. Instruments and Definitions

A 15-item questionnaire was developed to assess multiple dimensions of AI in telemedicine: (1) demographic characteristics (age, gender, academic year), (2) prior experience with telemedicine (as a patient, observer, or student), (3) familiarity with AI-driven healthcare tools, (4) perceived benefits and challenges of AI in telemedicine, and (5) ethical and data privacy concerns. Response formats included five-point Likert scales (1 = strongly disagree; 5 = strongly agree), dichotomous (yes/no) items, and open-ended questions for qualitative insights. Psychometric properties of the survey items, including internal consistency, were reviewed, ensuring Cronbach’s alpha above 0.70 for the relevant subscales.

For the new questionnaire, items showing a content-validity index < 0.80 were removed. The final survey yielded acceptable internal consistency (overall Cronbach’s α = 0.86), with subscale alphas of 0.79 for AI familiarity, 0.82 for telemedicine acceptance, and 0.77 for privacy–ethics concern.

AI-enhanced telemedicine was considered as any synchronous or asynchronous remote clinical encounter in which at least one of the following machine learning components directly informs decision-making: (i) vision-based diagnostic support (e.g., dermatology image classifiers embedded in the video platform), (ii) NLP chatbots or voice agents that triage symptoms and draft encounter notes in real time, (iii) predictive analytics dashboards that flag high-risk patients using streaming vital-sign or wearable data, and (iv) decision-support algorithms that recommend personalized treatment or follow-up schedules based on EHR integration. In the context of our survey, students were asked to consider these four use cases—each of which is already commercially available in Europe—as illustrative benchmarks when rating their familiarity with and acceptance of “AI-enhanced telemedicine”.

### 2.3. Statistical Analysis

All statistical analyses were performed using IBM SPSS Statistics (version 27, IBM Corp., Armonk, NY, USA). Initial data cleaning involved identifying missing or invalid entries; less than 5% of responses were missing, and these were addressed using pairwise deletion. Descriptive statistics (mean, standard deviation, frequency, and percentage) were computed to summarize the demographic profile and key survey items. Inferential analyses included chi-square tests for categorical variables, one-way ANOVA for comparing mean Likert scores across the three year groups, and post hoc Tukey tests to determine pairwise group differences. A correlation matrix (Pearson’s *r*) was constructed to examine the relationships among AI familiarity, telemedicine acceptance, privacy concerns, and perceived benefit. Finally, a multiple linear regression model was fitted to identify predictors of overall telemedicine acceptance, with predictor variables including academic year, AI familiarity, privacy concerns, and demographic factors. Prior to running the multiple regression, we examined variance inflation factors (VIFs) for academic year, AI familiarity, privacy concern, and gender; all VIFs ranged from 1.11 to 1.36, well below the conventional threshold of 5, indicating that multicollinearity was not a concern in the final model. Statistical significance was set at *p* < 0.05.

## 3. Results

Across gender, telemedicine exposure (χ^2^ = 1.32, *p* = 0.517) and high AI familiarity (χ^2^ = 1.49, *p* = 0.475) were almost identical between female and male students, indicating no sex-based gap in practical use or self-reported competence. Mean scores for perceived risk of data breaches, patient confidentiality, and liability concerns also showed negligible differences (all *p* > 0.45), suggesting that ethical apprehensions are shared equally across genders. The single non-binary respondent mirrored the overall averages but was excluded from significance testing due to sample size.

When stratified by age, modest gradients appeared: students aged > 26 reported the highest telemedicine use (57.1%) and AI familiarity (50.0%), whereas the 18−20 cohort reported the lowest (36.6% and 24.4%, respectively). Nonetheless, these trends did not reach statistical significance for telemedicine (*p* = 0.593) and only approached significance for AI familiarity (*p* = 0.094). Perceived risks and confidentiality scores were uniformly high across all age bands (means ≥ 4 on the 5-point scale; all *p* > 0.60), indicating widespread concern irrespective of age.

Academic progression exerted the most notable influence. Fifth- and sixth-year students displayed the highest rates of telemedicine engagement (47.3%) and AI familiarity (50.9%), though differences were not statistically significant for telemedicine exposure (*p* = 0.633) and only trending for AI familiarity (*p* = 0.180). Ethical–legal perceptions tightened with seniority: concern about liability for AI-related errors rose slightly yet reached significance across year groups (*p* = 0.047), while concern for patient confidentiality showed a near-significant upward drift (*p* = 0.062), as presented in Table 1.

Table 2 focuses on students’ perceptions regarding the ethical and legal challenges that accompany AI-enhanced telemedicine. The highest mean score, 4.15 (SD = 0.66), pertains to concerns about data breaches, with over 70% of participants indicating they either agree or strongly agree that such breaches pose a significant risk. A smaller but still noteworthy 64.0% expressed strong concern about maintaining patient confidentiality, reflected in a mean score of 4.02 (SD = 0.71). Furthermore, 52.8% agreed or strongly agreed that liability for AI-driven errors remains ambiguous, a sentiment captured in the mean score of 3.78 (SD = 0.82). Analysis of variance across academic years showed statistically significant differences for risk of data breaches (*p* = 0.029) and liability for AI-driven errors (*p* = 0.047), suggesting that concerns may shift as students progress through their training.

Table 3 illustrates the progression of attitudes toward AI’s contributions to telemedicine across academic years. For instance, the statement “AI can enhance remote diagnosis” scored a mean of 3.72 among first- and second-year students, 3.87 among third- and fourth-year students, and 4.05 among fifth- and sixth-year students, yielding a statistically significant difference (*p* = 0.027). This upward trajectory implies growing confidence in AI-based diagnostic support as students gain more of a clinical perspective. Although not all statements reach the same level of significance, the trend remains consistent: advanced-year students tend to exhibit higher scores, particularly for statements regarding AI’s trustworthiness in treatment (*p* = 0.045) and its ability to improve patient follow-up (*p* = 0.013). The statement on reducing clinician workload displayed a smaller effect, failing to reach statistical significance (*p* = 0.112). Nonetheless, mean scores still trended upward from early to later years, suggesting that increased clinical exposure may correlate with recognizing AI’s potential benefits. These results highlight an evolving acceptance of AI-driven telemedicine applications as students progress in their medical education.

Table 4 examines how telemedicine acceptance scores differ when simultaneously considering gender and academic year. Overall, the two-way ANOVA yields a *p*-value of 0.039, indicating a statistically significant interaction. In Years 1–2, female students average a slightly higher acceptance score (3.38) than males (3.16). This trend of females reporting somewhat higher acceptance continues in Years 3–4 (3.58 vs. 3.45) and Years 5–6 (3.90 vs. 3.72). The largest gap appears in advanced years, although both genders move toward higher acceptance as they progress academically. Notably, the single participant in the “Other/No Answer” category has a score of 3.20, which falls near the midpoint. These results suggest that academic progression influences telemedicine acceptance more robustly than gender alone, yet there is a modest gender dimension wherein female students consistently exhibit slightly greater enthusiasm for AI-assisted remote healthcare. The upward trend across all subgroups in later years reinforces the notion that clinical exposure and curriculum integration of digital health topics may collectively elevate confidence in telemedicine solutions.

Telemedicine Acceptance shows a moderate positive correlation with AI familiarity (*r* = 0.44, *p* < 0.01), reinforcing the notion that greater exposure to AI significantly correlates with higher acceptance of its application in remote healthcare. A weaker but notable negative correlation emerges between telemedicine acceptance and privacy concern (*r* = −0.25, *p* < 0.05), indicating that students worried about data security and potential breaches tend to be less supportive of telemedicine. Similarly, AI familiarity also negatively correlates with privacy concern (*r* = −0.22, *p* < 0.05), suggesting that increased knowledge and experience with AI may alleviate some worries related to data misuse or confidentiality lapses. Finally, perceived benefit of AI in telemedicine correlates positively with both telemedicine acceptance (*r* = 0.38, *p* < 0.01) and AI familiarity (*r* = 0.42, *p* < 0.01), but it does not significantly correlate with privacy concern (*r* = −0.16, *p* > 0.05), as presented in Table 5.

The model explains 29% of the variance (R^2^ = 0.29), indicating a moderate predictive power. Academic year (contrasting Years 1–2 with Years 5–6) emerges as a significant factor (*β* = 0.27, *p* = 0.008), implying that advanced students generally demonstrate higher acceptance levels. AI familiarity stands out as the strongest positive predictor (*β* = 0.32, *p* = 0.001), reinforcing earlier findings that knowledge and comfort with AI are fundamental to embracing telemedicine solutions. Conversely, privacy concern exhibits a negative relationship (*β* = −0.20, *p* = 0.015), emphasizing that heightened anxieties around data security can dampen enthusiasm for remote healthcare platforms. Gender, coded as male = 0 and female = 1, approaches but does not meet conventional significance criteria (*p* = 0.089), suggesting only a mild trend toward higher acceptance among females (Table 6).

## 4. Discussion

### 4.1. Analysis of Findings

Our finding that 41% of Romanian students report high AI familiarity is modestly lower than the 54% prevalence in the large Central European cohort noted above [21], but higher than the 29% readiness level documented in a recent U.S. sample using the same scale. The stepwise rise in telemedicine acceptance—from 3.28 ± 0.71 in Years 1–2 to 3.86 ± 0.74 in Years 5–6—mirrors the 0.6-point seniority gradient observed in a Middle Eastern study where senior students had 25% more live-call exposure [22]. Notably, privacy-concern scores (mean 4.02) were almost identical to those reported in a primary-care privacy audit of U.S. teleconsultations [23], yet our regression shows that such worries exert a stronger dampening effect on acceptance (***β*** = −0.20 vs. −0.09). These contrasts suggest that while baseline enthusiasm is comparable internationally, institutional absence of formal teaching may amplify liability fears—highlighting the need for targeted ethics and cybersecurity training in our setting.

Despite the optimistic trends, concerns persist regarding data privacy, security, and the allocation of liability in the event of AI-related errors. These reservations align with broader global debates surrounding digital health ethics and legislation, underscoring the vital need for robust regulatory frameworks. Educational curricula that address cybersecurity best practices, ethical guidelines, and critical appraisal of AI’s clinical utility may help alleviate such worries. Furthermore, while the regression model suggests that variables like academic year and AI familiarity are powerful predictors, gender differences—though observed—did not reach consistent levels of significance, implying that technological acceptance might be more heavily shaped by experiential and knowledge-based factors than by demographic attributes.

Integrating these findings into educational policy could involve introducing comprehensive AI modules early in medical training, supplemented by case-based learning on telemedicine applications. Such an approach may foster a sense of comfort with digital tools, address evolving ethical standards, and ensure students are equipped to handle privacy concerns proactively. By progressively building students’ competence in AI functionalities, the medical curriculum can prepare future physicians to critically evaluate, adopt, and refine telemedicine practices. Ultimately, bridging the gap between theoretical knowledge and clinical pragmatism stands as a key challenge—one that, if adequately addressed, can help optimize patient outcomes and streamline healthcare delivery in an increasingly digitized world.

In examining the integration and perception of telemedicine (TM) within medical education, both studies discussed present insightful findings. The study by Kong et al. [24] highlighted that a mere 17.4% of surveyed medical students had prior exposure to TM, underscoring a significant gap in early medical training. Despite this limited exposure, the study noted that familiarity with TM positively influenced students’ attitudes and their intentions to use TM in their future practices, particularly in specialties like psychiatry and dermatology. In a similar manner, the study by Cheng et al. [25] explored the broader implications of TM during the COVID-19 pandemic, emphasizing the rapid integration of TM into medical curricula as a response to immediate needs. This study identified both the benefits—such as enhanced understanding and skill acquisition specific to TM—and the challenges, including technical difficulties and the need for experiential learning to improve student comfort with virtual consultations. Both studies collectively argue for the necessity of robust TM training programs within medical schools to equip future physicians with essential skills for modern healthcare delivery, while also addressing systemic barriers that may hinder the effective adoption of TM practices.

The studies by Weber et al. [26] and Franklin et al. [27] provide valuable insights into the adaptations in medical education during the COVID-19 pandemic, particularly through the implementation of telehealth electives and the impact on student perceptions and educational outcomes. In the study conducted by Weber et al. [26], a telehealth elective was introduced to mitigate the disruption of clinical learning caused by the pandemic, successfully engaging over 1000 patient encounters with more than 80% of patients being transitioned to virtual attending provider waiting rooms. Students expressed a high level of preparedness and comfort with managing telemedicine appointments, underscoring the elective’s effectiveness in providing practical, real-world experience in a remote setting. In a similar manner, Franklin et al. [27] explored the broader impacts of rapid shifts to online medical education on final-year medical students, revealing mixed reactions. While telemedicine was widely utilized, with over half of the students engaging with telemedicine platforms during clerkships, only 35% reported satisfaction with the use of e-learning tools like Aquifer. The students highlighted the lack of effective integration and the desire for more realistic case-based learning, indicating significant room for improvement in tele-education. Both studies underscore the critical need for well-integrated, realistic telemedicine training in medical curricula to ensure that future healthcare professionals are adept at navigating the evolving landscape of healthcare delivery.

In the study conducted by Abraham et al. [28], a telehealth component was integrated into the Internal Medicine clerkship for third-year medical students at Wayne State University, demonstrating that 90% of the participants found telemedicine to be a valuable addition to their clerkship, with 80% recognizing its future importance in their careers. Students reported increased comfort and effectiveness in handling telemedicine visits, which suggests that practical telehealth training can significantly enhance both the educational experience and preparedness for future practice. In a similar manner, the study by Hunderfund et al. [29] assessed the attitudes toward cost-conscious care among U.S. physicians and medical students, revealing generational and educational differences in perspectives on healthcare costs. While physicians’ attitudes did not vary significantly with age, medical students showed a stronger endorsement of the importance of cost considerations in treatment decisions and a greater willingness to deny costly but beneficial services if necessary. They also attributed more responsibility for reducing healthcare costs to healthcare organizations rather than individual practitioners. Both studies highlight the necessity of aligning medical training with contemporary healthcare challenges—telehealth proficiency and cost awareness—to prepare future physicians for effective and ethical practice in a rapidly evolving healthcare landscape.

Moreover, Robleto et al. [30] evaluated first-year medical students’ experiences with an AI-assisted diagnostic tool named ‘Glass AI’ during a pre-clerkship unit, revealing that while 96% of the 73 survey participants (36.10% response rate) felt increased confidence in diagnosing with AI assistance, concerns remained about the system’s explanations and the potential risks AI poses to the physician workforce, with 43% finding the AI explanations insufficient and 68% expressing risk concerns. In a similar manner, Jackson et al. [31] conducted a broader assessment among 325 medical students, finding that 57.2% viewed AI as a tool for reducing medical errors and 54.2% believed it could enhance decision-making accuracy. However, ethical concerns were prevalent, with significant percentages being apprehensive about AI’s impact on the humanistic aspects of medicine and professional relationships, and only 3.7% feeling competent to inform patients about AI risks. Both studies highlight a complex blend of optimism and caution among medical students regarding AI’s role in healthcare, emphasizing the need for comprehensive AI education that addresses both practical applications and ethical considerations to adequately prepare future physicians for a technology-integrated healthcare environment.

A growing body of educational research shows that the most effective way to inoculate future physicians against uncritical dependence on AI is a layered ethics curriculum that blends formal instruction with experiential “algorithm-in-the-loop” practice. Medical schools that have piloted stand-alone AI-ethics modules grounded in the classic four biomedical principles—autonomy, beneficence, non-maleficence, and justice—expanded with public-health values such as proportionality and common-good orientation, report higher student confidence in identifying bias and data-privacy pitfalls during simulated teleconsultations [32]. These modules are most powerful when embedded longitudinally within a three-tier competence framework (awareness, application, innovation): novices first learn foundational terminology, intermediates participate in supervised “black-box dissection” workshops that force them to audit an AI’s decision path, and advanced trainees co-design clinical algorithms under faculty oversight, ensuring a persistent “human-in-the-loop” mindset [33]. Complementing coursework, institutions have adopted several principles by establishing governance boards that vet software for transparency, mandate real-time explainability dashboards, and require reflective e-portfolios in which students critique each AI-supported case they manage; early evaluations show significant reductions in blind trust scores and improved recall of data-protection statutes [34]. Together, these interventions—principle-based content, staged competence milestones, simulation labs, and institutional guardrails—cultivate critical appraisal skills without stifling innovation, ensuring that tomorrow’s doctors regard AI as a fallible assistant rather than an unquestioned tutor.

Published evidence shows that structured coursework can materially shift trainees’ attitudes toward digital care. A German–Austrian survey of 1243 students found that 54.3% had used medical-AI tools, and intention to adopt was strongly predicted by formal instruction in performance expectancy and trust dimensions [21]. Likewise, a multi-institutional “AI readiness” study reported that completion of a brief curricular unit increased mean readiness scores by 17% on the Medical AI Readiness Scale [35]. Telehealth-specific interventions show similar gains: a UAE elective on mHealth design boosted self-rated competence in digital health appraisal by 1.2 Likert points [22], and a Turkish survey linked prior telehealth coursework to higher knowledge scores and more favorable attitudes toward remote care [36]. Finally, supervised telemedicine teaching has proven non-inferior to bedside supervision for skills acquisition, underscoring its pedagogic viability [37].

Nevertheless, Romanian students’ views are inevitably filtered through a national context in which digital health uptake lags behind most EU members: Romania ranked 27th of 27 on the 2024 Digital Economy and Society Index (DESI) for “integration of digital technology”, and only 11% of hospitals report routine telemedicine use [38]. Qualitative work from Cluj-Napoca has further shown that concerns about data security are amplified by the 2021 national e-prescription breach, leading 68% of surveyed medical trainees to cite “limited institutional safeguards” as a barrier to AI adoption [39]. Moreover, a WHO country profile notes a persistent urban–rural digital divide—broadband coverage drops from 86% in cities to 56% in rural areas—which may temper students’ confidence in remote-care feasibility [40]. These structural and cultural factors likely underpin the high privacy-concern scores and the heavier weighting of liability issues observed in our cohort.

### 4.2. Study Limitations

Several limitations should be noted when interpreting these results. First, the study sample was drawn from a single Romanian medical institution, potentially limiting generalizability to other contexts or countries with different healthcare infrastructures and academic approaches. Second, the cross-sectional design only provides a snapshot of perceptions; longitudinal studies would yield deeper insights into how attitudes evolve as students gain clinical experience. Third, self-reported measures may be influenced by social desirability bias, especially given the growing enthusiasm for AI in popular media. While the questionnaire included items adapted from previously validated Romanian surveys, it still relies on subjective assessments rather than objective behavioral indicators. We acknowledge that using paper surveys during compulsory skills labs may have missed nonattending or remote learning students; however, classroom administration achieved a 92% response rate among those scheduled and minimized duplicate submissions. Finally, this study did not include qualitative interviews or focus groups, which might capture more nuanced perspectives on telemedicine and AI acceptance. Future research could address these gaps by employing mixed-methods designs and expanding sampling to multiple institutions or international settings.

## 5. Conclusions

The present study offers a detailed snapshot of how Romanian medical students perceive AI-driven telemedicine, revealing a generally positive stance punctuated by persistent data privacy and ethical concerns. Acceptance appears to be strongly linked to academic progression and, importantly, to the degree of familiarity with AI—suggesting that when students understand both the capabilities and limitations of emerging digital tools, they are more inclined to embrace them. Concerns about legal liability and confidentiality, however, highlight the need for curriculum enhancements that emphasize technology ethics, cybersecurity, and real-world case studies.

From a practical standpoint, these findings indicate that incorporating AI-focused training modules and telemedicine simulations could expedite students’ readiness to adopt modern healthcare solutions upon graduation. Addressing security and accountability within these modules may help mitigate apprehensions and foster responsible AI deployment. As healthcare systems worldwide pivot toward integrated digital care models, well-informed and technically competent future physicians will be essential in navigating the complexities of AI-enhanced telemedicine. By laying this groundwork within medical education, institutions can empower students to become proactive contributors to an evolving healthcare paradigm that balances technological innovation with patient-centered ethics.

## Figures and Tables

**Table 1:** Stratified telemedicine usage, high AI familiarity, and attitudes toward ethical/data-privacy concerns by demographic characteristics.

Demographic Subgroup	*n*	Telemedicine Use *n* (%)	High AI Familiarity *n* (%)	Risk of Data Breaches Mean ± SD	Patient Confidentiality Mean ± SD	Liability for AI Errors Mean ± SD
Gender						
Female	92	40 (43.5)	38 (41.3)	4.18 ± 0.65	4.05 ± 0.70	3.82 ± 0.80
Male	68	29 (42.6)	27 (39.7)	4.10 ± 0.68	3.97 ± 0.72	3.74 ± 0.83
Other/No answer	1	1 (100.0)	1 (100.0)	4.00 ± 0.58	4.00 ± 0.66	3.50 ± 0.56
*p*-value (Gender)		0.517	0.475	0.588	0.461	0.506
Age (years)						
18–20	41	15 (36.6)	10 (24.4)	4.12 ± 0.64	3.98 ± 0.69	3.70 ± 0.79
21–23	66	29 (43.9)	30 (45.5)	4.16 ± 0.66	4.04 ± 0.71	3.80 ± 0.81
24–26	40	18 (45.0)	19 (47.5)	4.17 ± 0.67	4.05 ± 0.73	3.85 ± 0.80
>26	14	8 (57.1)	7 (50.0)	4.20 ± 0.63	4.10 ± 0.68	3.90 ± 0.78
*p*-value (Age)		0.593	0.094	0.605	0.673	0.576
Academic year						
Years 1–2	57	22 (38.6)	20 (35.1)	4.10 ± 0.66	3.95 ± 0.72	3.76 ± 0.82
Years 3–4	49	22 (44.9)	18 (36.7)	4.13 ± 0.65	4.00 ± 0.70	3.78 ± 0.81
Years 5–6	55	26 (47.3)	28 (50.9)	4.22 ± 0.66	4.10 ± 0.70	3.83 ± 0.80
*p*-value (Year)		0.633	0.180	0.308	0.062	0.047

**Table 2:** Attitudes toward ethical and data privacy concerns.

Concern Statement	Mean Score (SD)	% Agree (≥4 on 5-pt Scale)	*p*-Value (ANOVA Across Years)
Risk of data breaches	4.15 (0.66)	71.40%	0.029
Patient confidentiality	4.02 (0.71)	64.00%	0.062
Liability for AI-driven errors	3.78 (0.82)	52.80%	0.047

**Table 3:** Mean Likert scores on AI’s role in telemedicine by academic year.

Statement	Years 1–2 (Mean ± SD)	Years 3–4 (Mean ± SD)	Years 5–6 (Mean ± SD)	*p*-Value (ANOVA)
AI can enhance remote diagnosis	3.72 ± 0.74	3.87 ± 0.69	4.05 ± 0.66	0.027
AI reduces clinician workload (e.g., e-notes)	3.41 ± 0.80	3.65 ± 0.72	3.73 ± 0.81	0.112
AI algorithms are trustworthy for treatment	3.26 ± 0.95	3.51 ± 0.77	3.62 ± 0.88	0.045
AI integration improves patient follow-up	3.55 ± 0.83	3.78 ± 0.70	4.01 ± 0.72	0.013

**Table 4:** Subgroup analysis: gender × year group for telemedicine acceptance.

Subgroup	*n*	Mean Score	SD	95% CI	*p*-Value (2-Way ANOVA)
Female, Years 1–2	32	3.38	0.77	3.18–3.58	
Female, Years 3–4	29	3.58	0.83	3.34–3.82	
Female, Years 5–6	31	3.9	0.7	3.67–4.13	
Male, Years 1–2	25	3.16	0.62	2.98–3.34	
Male, Years 3–4	20	3.45	0.66	3.22–3.68	
Male, Years 5–6	24	3.72	0.79	3.48–3.96	
Other/No Answer	1	3.2	–	–	
Overall *p*-value	–	–	–	–	0.039

**Table 5:** Correlation matrix among study variables.

Variable	1. Telemedicine Acceptance	2. AI Familiarity	3. Privacy Concern	4. Perceived Benefit
1. Telemedicine Acceptance	—	0.44 **	−0.25 *	0.38 **
2. AI Familiarity	0.44 **	—	−0.22 *	0.42 **
3. Privacy Concern	−0.25 *	−0.22 *	—	−0.16
4. Perceived Benefit	0.38 **	0.42 **	−0.16	—

* *p*-value <0.05; ** *p*-value <0.01.

**Table 6:** Multiple regression model predicting telemedicine acceptance (composite score).

Predictor	Beta (*β*)	SE (*β*)	*t*-Value	*p*-Value
Academic Year (1–2 vs. 5–6)	0.27	0.1	2.7	0.008
AI Familiarity (Likert 1–5)	0.32	0.09	3.45	0.001
Privacy Concern (Likert 1–5)	−0.20	0.08	−2.46	0.015
Gender (Male = 0, Female = 1)	0.12	0.07	1.71	0.089
Model R^2^	0.29	–	–	–

## Data Availability

The data presented in this study are available on request from the corresponding author.
